# Supplementary material for: Evolutionary persistence of a highly prevalent multicopy mitochondrial-derived nuclear insertion (Mega-NUMT) in Neotropical Drosophila flies
Source: Mol Biol Evol. 2026 Sep 1;43(9):msag227. doi: 10.1093/molbev/msag227 (PMC13565137; doi:10.1093/molbev/msag227)
Supplement: Supplementary file 1 [file msag227_supplementary_data.pdf]

Supplementary material

Table S1. List of primer sets used for qPCR and RFLP-PCR. Table lists the primer sequence and the according annealing temperature for each primer set used in PCR as well as the corresponding amplicon size.

Locus	Primer name	5'-3' primer sequence	Target	Tm	Fragment length	experiment
RPS17	qRp 17 melwin 1 F	GCCGTCTGCGTCACTCC	Fly control gene	60°C	106bp	qPCR
RPS17	qRp 17 melwil1_R	GCTTCAACATCTCCTTGGTATCG	Fly control gene	60°C	106bp	qPCR
CO3	coxSNP_1	GACATTTTGTTGATGTAGTT	alpha	52°C	121bp	discPCR
CO3	coxSNP_2	GACATTTTGTTGATGTAGTG	beta	52°C	121bp	discPCR
CO3	coxSNP_uni	TAGACCTTATGATTGGAAGT	Universal reverse mitotype	52°C	121bp	discPCR
CO1	CO1-F	TTTAATTTTACCTGGATTTGG		56°C	788bp	RFLP-PCR
CO1	CO1_wob-F	TTTAATTTTACCWGGATTTGG		56°C	788bp	RFLP-PCR
CO1	CO1_univ-R	ATTCAGAATATCTATGTTCAG		56°C	788bp	RFLP-PCR
CO1	CO1_wm-F	TTATTTACAGGATTAACTTTAAATGC		56°C	705bp	RFLP-PCR
CO1	CO1_wm-R	ACAGAAGGTTCATTAATTTCATC		56°C	705bp	RFLP-PCR
CO2	CO2-F	ATGTCTACATGAGCTAATTTAGGT		56°C	323bp	RFLP-PCR
CO2	CO2-R	TAACTTCAGTATCATTGGTGACC		56°C	323bp	RFLP-PCR
CO3	CO3-F	TATAATTCAATCTTATGTATTTGCAG		55°C	841bp	RFLP-PCR
CO3	CO3-R	GATTATTCCTTTATATTACAATTTACT		55°C	841bp	RFLP-PCR
ND2	ND2-F	CTTCYATTAATCATTTAGGATGAATATTAAGYGC		65°C	357bp	RFLP-PCR
ND2	ND2-R	AAGCTGAATAACAAATTCGTAARTAAAAAAATAAAG		65°C	357bp	RFLP-PCR

Table S2. F-test statistics to test differences in relative copy number of β-mitochondria and α-NUMT across different parameters: tissue, sex, age and combinations of those. The main results are shown in Figure 2. Df: degrees of freedom; sq: square.

**Cox1-α**	**Df**	**Sum Sq**	**Mean Sq**	**F Value**	**P-value (>F)**
**Tissue**	4	2.43	0.6070	1.728	0.150
**Sex**	1	0.77	0.7713	2.195	0.142
**Age**	1	0.00	0.0001	0.000	0.986
**Tissue:Sex**	4	0.10	0.0249	0.071	0.991
**Tissue:Age**	4	0.14	0.0343	0.097	0.983
**Sex:Age**	1	0.17	0.1660	0.472	0.493
**Tissue:Sex:Age**	4	1.05	0.2620	0.746	0.563
**Residuals**	100	35.13	0.3513		
**Cox2-β**	**Df**	**Sum Sq**	**Mean Sq**	**F Value**	**P-value (>F)**
**Tissue**	4	26.525	6.631	27.694	1.76e-15***
**Sex**	1	1.129	1.129	4.714	0.0323*
**Age**	1	0.011	0.011	0.044	0.8343
**Tissue:Sex**	4	1.145	0.286	1.195	0.3175
**Tissue:Age**	4	0.447	0.112	0.467	0.7598
**Sex:Age**	1	1.596	1.596	6.666	0.0113*
**Tissue:Sex:Age**	4	1.269	0.317	1.325	0.2660
**Residuals**	100	23.945	0.239		

Table S3. Genome assembly information across different assembly methods. Unfiltered refers to raw reads being used directly in Nextdenovo to produce the genome assembly. Phred filters refer to the priority of quality of the reads when subsampling reads to the marked coverage (X40, X50). Length sets priority in read length when subsampling reads. Two variations (noted with "+ assembly ref") included the unfiltered assembly as reference for the subsampling. The second column shows how many reads were left after subsampling, and the third column indicates the total number of base pairs in the dataset assembled. Quast was used to assess assembly size, contigs, N50 and the size of the largest contig. BUSCO was used to assess genome completeness based on single-copy genes.

O11	Reads	bp	Coverage	Assembly size	Contigs	N50	BUSCO	Largest contig
unfiltered	1 376 601	19 454 247 303	77	251 478 094	188	28 092 762	C:97.3%[S:96.3%,D:1.0%],F:1.1%, M:1.6%,n:3285	35 123 609
Phred X40	641 549	10 000 005 368	40	224 185 510	96	16 811 753	C:97.2%[S:96.4%,D:0.8%],F:1.1%, M:1.7%,n:3285	29 505 846
Phred X40 + assembly ref	472 844	10 000 013 032	40	225 554 080	107	15 921 554	C:97.5%[S:96.6%,D:0.9%],F:1.0%, M:1.5%,n:3285	31 708 369
Phred X50	827 899	12 500 020 744	50	231 244 016	111	18 692 465	C:97.6%[S:96.7%,D:0.9%],F:1.2%, M:1.2%,n:3285	33 659 176
Length X40	312 151	10 000 014 293	40	235 601 215	126	13 106 850	C:97.1%[S:96.3%,D:0.8%],F:1.1%, M:1.8%,n:3285	29 628 043
Length X40 + assembly ref	296 871	10 000 014 326	40	231 875 968	113	25 388 691	C:97.2%[S:96.3%,D:0.9%],F:1.1%, M:1.7%,n:3285	37 599 843
Length X50	827 899	12 500 012 596	50	239 343 741	128	16 562 743	C:97.2%[S:96.4%,D:0.8%],F:0.9%, M:1.9%,n:3285	39 187 662

Table S4. Polishing iterations of the genome assembly, accounting for the number of fixes that each method has produced, and the final BUSCO score. They are ordered in the order in which they were done over the assembly Pepper -> Pilon1 -> Pilon2 -> Pilon3. The assembly polished by two rounds of pilon was selected as the optimal genome assembly, to avoid introducing errors.

Polishing method	Insertions	Deletions	SNPs	Total fix	BUSCO
PEPPER	68	1719	4071	4071	C:97.6%[S:96.7%,D:0.9%],F:1.2%,M:1.2%,n:3285
Pilon 1	139108	37585	0	176693	C:99.0%[S:98.1%,D:0.9%],F:0.3%,M:0.7%,n:3285
Pilon 2	1261	1719	0	2980	C:99.0%[S:98.1%,D:0.9%],F:0.3%,M:0.7%,n:3285
Pilon 3	176	535	0	711	C:99.0%[S:98.1%,D:0.9%],F:0.3%,M:0.7%,n:3285

Table S5. **Genes and pseudogenes of α-NUMTs in the genome assembly of *D. paulistorum* O11.** Each row represents one NUMT copy found in the genome assembly, while the columns represent the 13 mitochondrial genes of the *D. paulistorum* mitochondrial genome (listed below). Green = complete gene, yellow = pseudogene, grey = absence of the gene. *ND2*: NADH dehydrogenase subunit2, *CO1*: cytochrome c oxidase subunit I, *CO2*: cytochrome c oxidase subunit II, *ATP8*: ATP synthase F0 subunit 8, *ATP6*: ATP synthase F0 subunit 6, *CO3*: cytochrome c oxidase subunit III, *ND3*: NADH dehydrogenase subunit 3, *ND5*: NADH dehydrogenase subunit 5, *ND4*: NADH dehydrogenase subunit 4, *ND4L*: NADH dehydrogenase subunit 4L, *ND6*: ND6 dehydrogenase subunit 6, *cytB*: Cytochrome b, *ND1*: NADH dehydrogenase subunit 1.

	ND2	*CO1*	*CO2*	*ATP8*	*ATP6*	*CO3*	*ND3*	*ND5*	*ND4*	*ND4L*	*ND6*	*cytB*	*ND1*
NUMT 1													
NUMT 2													
NUMT 3													
NUMT 4													
NUMT 5													
NUMT 6													
NUMT 7													
NUMT 8													
NUMT 9													
NUMT 10													
NUMT 11													
NUMT 12													

Table S6. List of fly lines used in the study belonging to willistoni group species. Abbreviation: NDSSC - National Drosophila Species Stock Center

Species	Subspecies	Line	Locality	Source/Collector	Date
D. equinoxialis		FS	Fort Sherman, Colon, Panama	Egon Bartel	2002
D. insularis		MASS-B-SL	St Lucia	Jeff Powell	2006
D. tropicalis		SS	San Salvador, El Salvador	NDSSC: 14030-0801.01/ William Heed	1955
D. willistoni		APA8-2	Veracruz, México	Joana Silva	1998
		FG168	Saül, French Guiana, France	Wolfgang Miller & Aurélie Hua-Van	2014
D. paulistorum	*Amazonian*	A28	Belém, PA, Brazil	Lee Ehrman	1952
		FG572	Saül, French Guiana, France	Wolfgang Miller & Aurélie Hua-Van	2015
		FG16	Saül, French Guiana, France	Wolfgang Miller & Aurélie Hua-Van	2018
		FG18	Saül, French Guiana, France	Wolfgang Miller & Aurélie Hua-Van	2018
	Andean-Brazilian	MS	Mesitas, Colombia	Lee Ehrman	1962
		Yellow	Copan, Honduras	NDSSC: 14030-0771.02	1960
	Centro american	C2	Lancetilla, Honduras	Lee Ehrman	1954
		White	Lancetilla, Honduras	NDSSC: 14030-0771.03	1960
	Interior	L1	Llanos, Colombia	Lee Ehrman	1958
	Orinocan	O11	Georgetown, Guyana	Lee Ehrman	1957
		RP	Ribeirão Preto, SP, Brazil	Ana Laurer Garcia	1995
		POA1	Porto Alegre, RS, Brazil	Ana Laurer Garcia	2003
		TP37	Pousada Triunfo, PE, Brazil	Claudia Rohde & Ana Laurer Garcia	2009
		FG103	Saül, French Guiana, France	Wolfgang Miller & Aurélie Hua-Van	2014
		FG111	Saül, French Guiana, France	Wolfgang Miller & Aurélie Hua-Van	2014
		FG295	Saül, French Guiana, France	Wolfgang Miller & Aurélie Hua-Van	2014
	Transitional	SM	Santa Marta, Colombia	Lee Ehrman	1956
D. nebulosa		NEB	Palmira, Colombia	NDSSC: 14030-0761.00/ William Heed	1955

Table S7. Mega-NUMT prevalence in *D. paulistorum* from recent collections in French Guiana

**Line Origin**[1]	**Collection Date**	**Latitude**	**Longitude**	**Mega-NUMT**[2]	**Prevalence**
**Long-term stocks from 1960s**	*see* Table 1 and Table S6			**4/9**	**0.44**
**French Guiana, F**					
CNRS Camp Inselberg	June 2015	04˚05'16.73" N	52˚40'47.31"W	8/23	0.35
CNRS Camp Inselberg	Aug. 2019	04˚05'16.73" N	52˚40'47.31"W	4/8	0.50
CNRS Camp Pararé	May 2014	04˚02'16.28" N	52˚40'22.55"W	2/5	0.40
Route to Kaw	Sept. 2024	04˚33'34.27" N	52˚12'26.11"W	6/14	0.43
Saül, Chez Lulu	May 2014	03˚37'16.35" N	53˚12'41.62"W	8/15	0.53
Saül, Chez Lulu	August 2019	03˚37'16.35" N	53˚12'41.62"W	4/11	0.36
Maripasoula, Le terminus	May 2014	03˚38'09.18" N	54˚01'44.58"W	2/4	0.50
**French Guiana total:**	2014-2024			**34/80**	**0.43**

[1] Geographic origin of the established isofemale line.
[2] Number of isolines carrying a Mega-NUMT and number of total isolines tested are based on diagnostic double-peaks in chromatograms of direct *CO1* Sanger sequencing and cloning. For field samples from French Guiana, Mega-NUMT-typing was performed on multi-fly DNA from established isofemale lines at generation F1 and F2.

Table S8. Mega-NUMT-typing of the 80 isofemale lines collected between 2014 and 2024 at different localities in French Guiana (summarized in Figure 7). Mega-NUMT-typing was performed by *CO1* direct Sanger sequencing and/or quantitative RFLP-PCR for abundance and prevalence for samples with available data sets. After more than 10 years under lab conditions (highlighted in gray and tested again in 2026), only 7 out of the 16 originally MN-positively typed at F1/F2 lines have maintained the Mega-NUMT (43.75%), dropping from initial 51.6% (16/31) to 22.6% (7/31) under lab conditions.

French Guiana Iso-Line	Sampling Year	*D. paulistorum* Spp	Mito-type	α-NUMT detected in F1 by Sanger	α-NUMT detected in 2026 by Sanger	α-NUMT Abundance (%)# f/m	α-NUMT Prevalence
**Maripousala**							
FG127	2014	AM	ß1	-	-	0	
FG128	2014	AM	ß1	-	-	0	
FG219	2014	AM	ß1	Yes	-	ND	ND
FG238	2014	AM	ß1	Yes	NA	ND	ND
**CNRS Camp Pararé**							
FG248	2014	AM	ß1	-	-	0	
FG250	2014	AM	ß1	Yes	NA	57/62.7	ND
FG254	2014	AM	ß1	**Yes**	**Yes**	27.6/66.0	ND
FG256	2014	AM	ß1	-	-	0	
FG259	2014	AM	ß1	-	-	0	
**Saül**							
FG82	2014	OR	ß2	-	NA	0	0% (10/10)
FG89	2014	AM	ß1	Yes	-	ND	0% (0/40)
FG97	2014	OR	ß2	-	NA	0	0% (10/10)
FG103	2014	OR	ß2	Yes	-	ND	0% (0/40)
FG104	2014	OR	ß2	-	-	0	0% (10/10)
FG105	2014	OR	ß2	Yes	-	18.6/24.2	0% (19/20)
FG109	2014	OR	ß2	Yes	NA	ND	ND
FG111	2014	OR	ß2	**Yes**	**Yes**	10.3/11.8	55% (22/40)
FG113	2014	OR	ß2	**Yes**	**Yes**	6.8/12	80% (16/20)
FG160	2014	AM	ß1	-	NA	0	0% (10/10)
FG169	2014	AM	ß1	**Yes**	**Yes**	58.6/52.6	15% (3/20)
FG186	2014	AM	ß1	Yes	-	42.4/45.7	35% (7/20)
FG217	2014	AM	ß1	-	-	0	0% (10/10)
FG271	2014	AM	ß1	-	-	0	0% (10/10)
FG295	2014	OR	ß2	-	-	0	0% (0/40)
**Saül**							
FG-MS1	2019	AM	ß1	-	ND	0	
FG-MS2	2019	AM	ß1	-	ND	0	
FG-WS1	2019	AM	ß1	-	ND	0	
FG-WS2	2019	AM	ß1	-	ND	0	

FG-WS3	2019	AM	ß1	Yes	ND	50.8/49.7	100% (20/20)
FG-WS6	2019	OR	ß2	Yes	ND	NA	NA
FG-WS7	2019	OR	ß2	Yes	ND	49.7/54.5	50% (10/20)
FG-WS12	2019	OR	ß2	Yes	ND	NA	NA
FG-WB2	2019	AM	ß1	-	ND	0	
FG-WB3	2019	AM	ß1	-	ND	0	
FG-WB4	2019	AM	ß1	-	ND	0	

Camp CNRS Inselberg

FG508	2015	AM	ß1	Yes	NA	31/25.2	10% (2/20)
FG509	2015	AM	ß1	Yes	-	51.1/69.8	50% (10/20)
FG526	2015	AM	ß1	-	NA	0	
FG528	2015	AM	ß1	-	NA	0	
FG529	2015	AM	ß1	**Yes**	**Yes**	47.2/54.2	35% (7/20)
FG546	2015	AM	ß1	Yes	-	NA	NA
FG547	2015	AM	ß1	-	NA	0	
FG548	2015	AM	ß1	-	NA	0	
FG549	2015	AM	ß1	-	NA	0	
FG550	2015	AM	ß1	-	-	0	
FG559	2015	AM	ß1	**Yes**	**Yes**	39.7/52.4	80% (16/20)
FG569	2015	AM	ß1	Yes	NA	NA	NA
FG570	2015	AM	ß1	Yes	-	46.1/44.6	50% (10/20)
FG572	2015	AM	ß1	**Yes**	**Yes**	47.3/68.7	95% (19/20)
FG585	2015	AM	ß1	-	NA	0	
FG594	2015	AM	ß1	-	NA	0	
FG601	2015	AM	ß1	-	-	0	
FG602	2015	AM	ß1	-	-	0	
FG609	2015	AM	ß1	-	-	0	
FG614	2015	AM	ß1	-	NA	0	
FG617	2015	AM	ß1	-	-	0	
FG618	2015	AM	ß1	-	-	0	
FG620	2015	AM	ß1	-	-	0	

Camp CNRS Inselberg

FG-WI10	2019	AM	ß1	-	ND	0	
FG-WI14	2019	AM	ß1	Yes	ND	58.97/64.19	40% (8/20)
FG-WI17	2019	AM	ß1	Yes	ND	61.39/64.49	80% (16/20)
FG-WI21	2019	AM	ß1	-	ND	0	
FG-WI24	2019	AM	ß1	-	ND	0	
FG-WIP1	2019	AM	ß1	Yes	ND	41.04/71.59	75% (15/20)
FG-WIP2	2019	AM	ß1	-	ND	0	
FG-WIP3	2019	AM	ß1	Yes	ND	34.72/55.55	80% (16/20)

Road to Kaw

FG-A4	2024	AM	ß1	-	ND	0	
FG-A5	2024	AM	ß1	-	ND	0	
FG-A8	2024	AM	ß1	-	ND	0	
FG-A11	2024	AM	ß1	-	ND	0	

FG-A12	2024	AM	ß1	-	ND	0	
FG-A15.1	2024	AM	ß1	Yes	ND	NA	NA
FG-A16.1	2024	AM	ß1	Yes	ND	NA	NA
FG-A14	2024	AM	ß1	Yes	ND	NA	NA
FG-A21.1	2024	AM	ß1	Yes	ND	NA	NA
FG-A22	2024	AM	ß1	-	ND	0	
FG-A24	2024	AM	ß1	-	ND	0	
FG-A28	2024	AM	ß1	-	ND	0	
FG-A37	2024	AM	ß1	Yes	ND	NA	NA
FG-AB3	2024	AM	ß1	Yes	ND	NA	NA

Abbreviations: NA: not available, because the line was lost over the last 10 years in the laboratory; ND: not determined; Yes: presence of the α-NUMT detected by the presence of double peaks in *CO1* chromatograms, and/or by the presence of the diagnostic *Hin*dIII RFLP restriction site. Gray backgrounded lines: Isolines that were maintained for more than 10 years under laboratory conditions and recently *CO1*-barcoded by direct Sanger sequencing in pools of 4 females and 6 males (n=31). Bold: Lines that have maintained in 2026 their Mega-NUMTs under laboratory conditions, like drift and artificial selection. (-) Absence of the MN in direct Sanger reads.

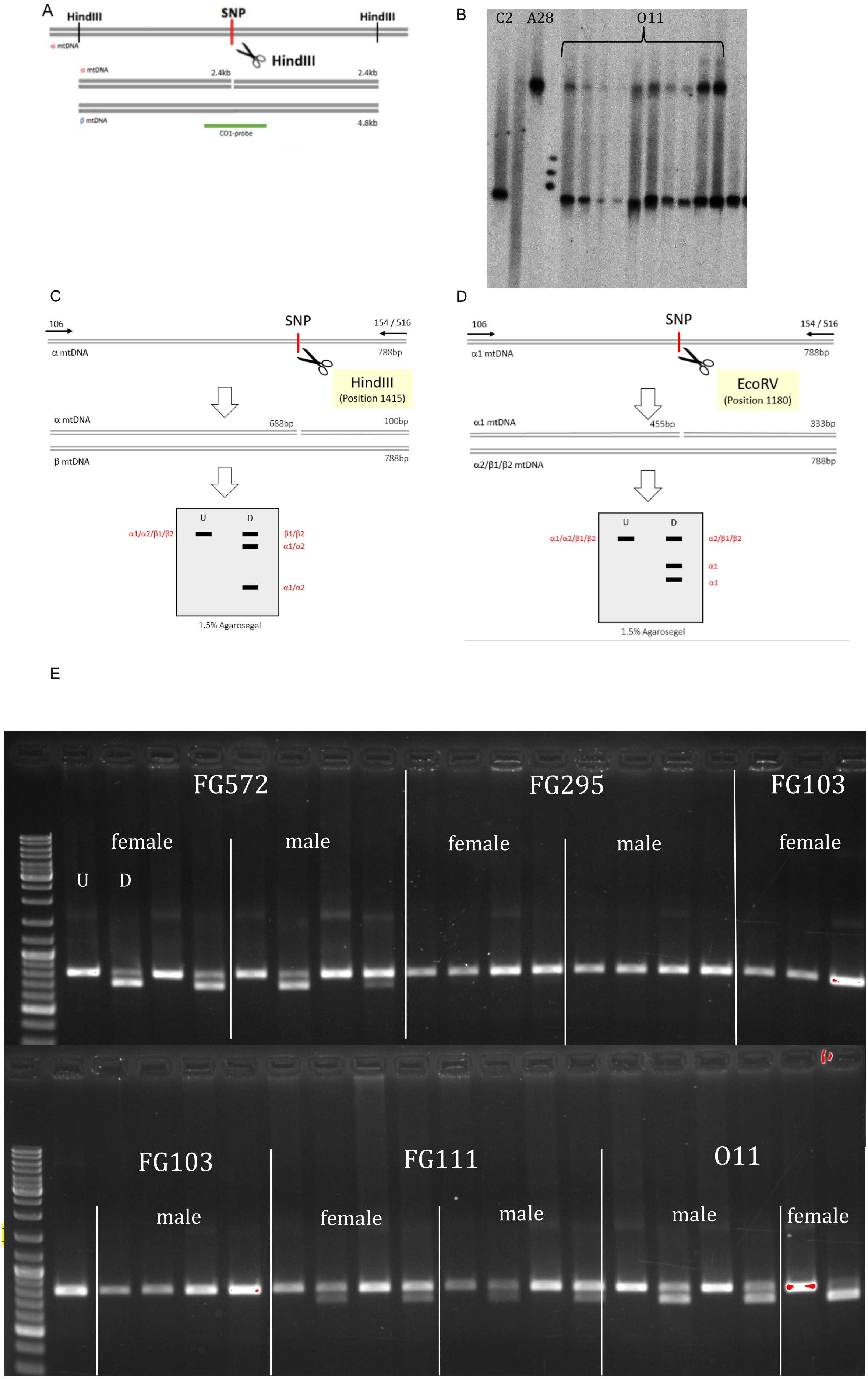

A
HindIII
SNP
HindIII
α mtDNA
HindIII
α mtDNA
2.4kb
2.4kb
β mtDNA
4.8kb
CO1-probe
B
C2
A28
O11
C
106
SNP
154 / 516
α mtDNA
788bp
HindIII
(Position 1415)
α mtDNA
688bp
100bp
β mtDNA
788bp
U
D
α1/α2/β1/β2
β1/β2
α1/α2
α1/α2
1.5% Agarosegel
D
106
SNP
154 / 516
α1 mtDNA
788bp
EcoRV
(Position 1180)
α1 mtDNA
455bp
333bp
α2/β1/β2 mtDNA
788bp
U
D
α1/α2/β1/β2
α2/β1/β2
α1
α1
1.5% Agarosegel
E
FG572
female
U
D
male
FG295
female
male
FG103
female
FG103
male
FG111
female
male
O11
male
female

F

Single Flies line FG572
(α2, ß1) – *COI, Hind*III

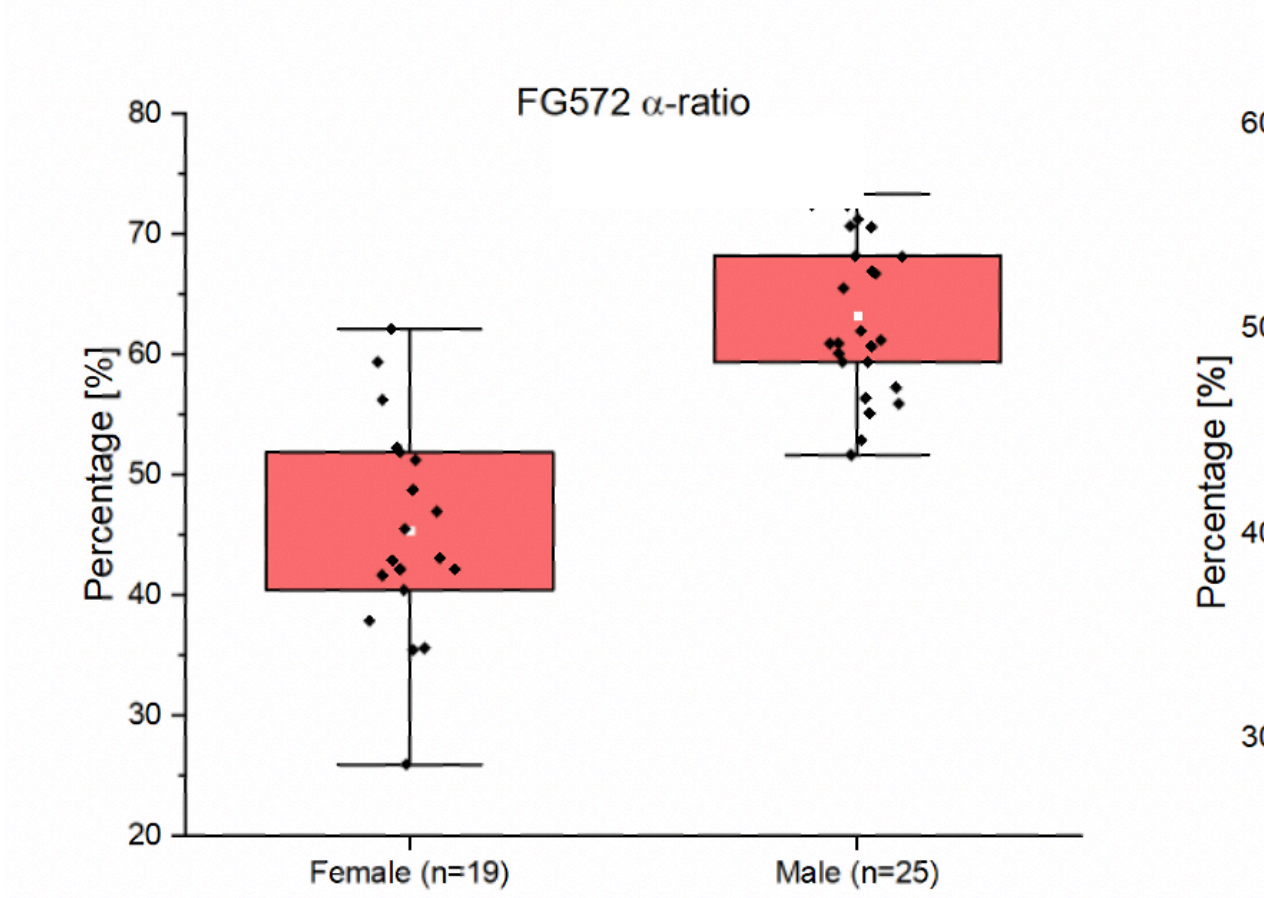

COI	α - Mean	SD	Transmission
**female (n=19)**	45.34	8.91	95%
**male (n=25)**	63.19	6.54	83%

Single Flies MS
(α1, α2) – *COI, Eco*RV

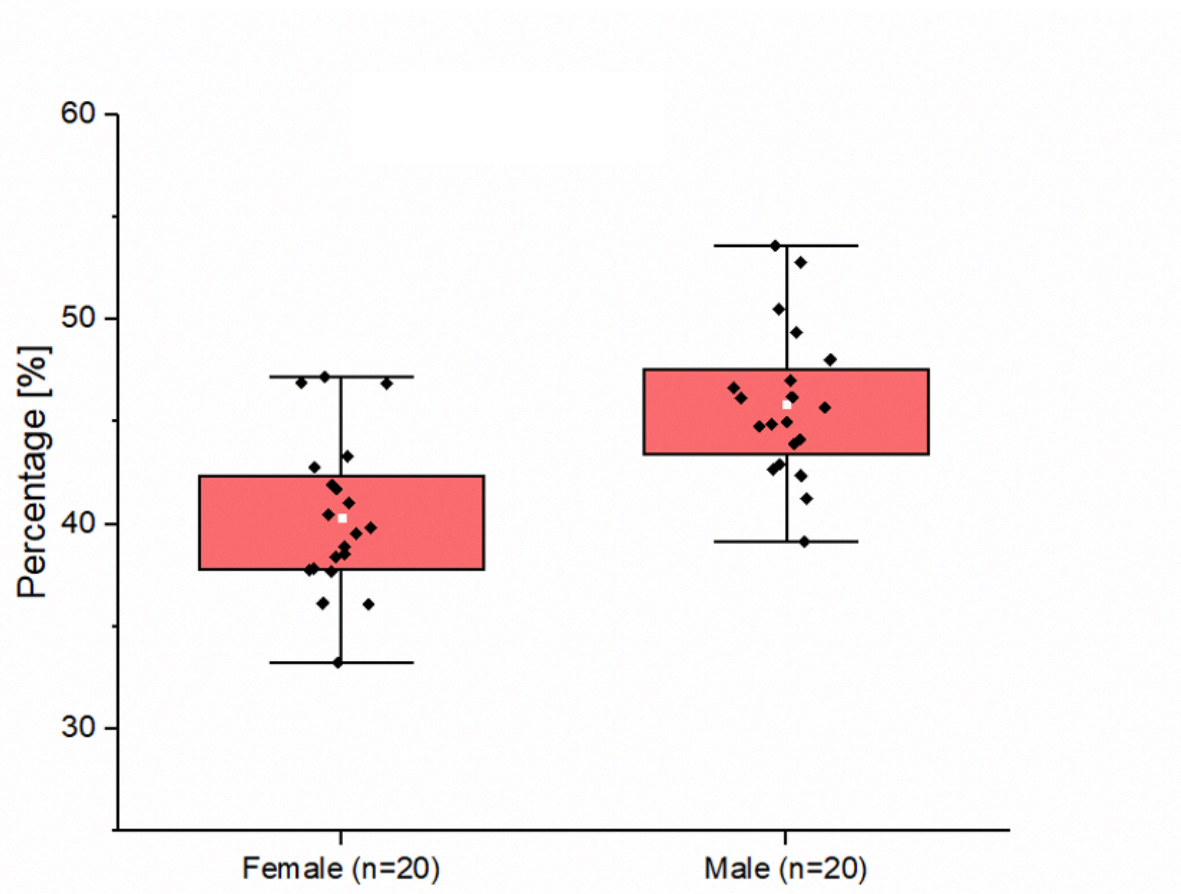

COI	α2 - Mean	SD	Transmission
**female (n=20)**	40.31	3.76	100%
**male (n=20)**	45.86	3.67	100%

Single Flies FG16
(α2, ß1) – *COI, Hind*III

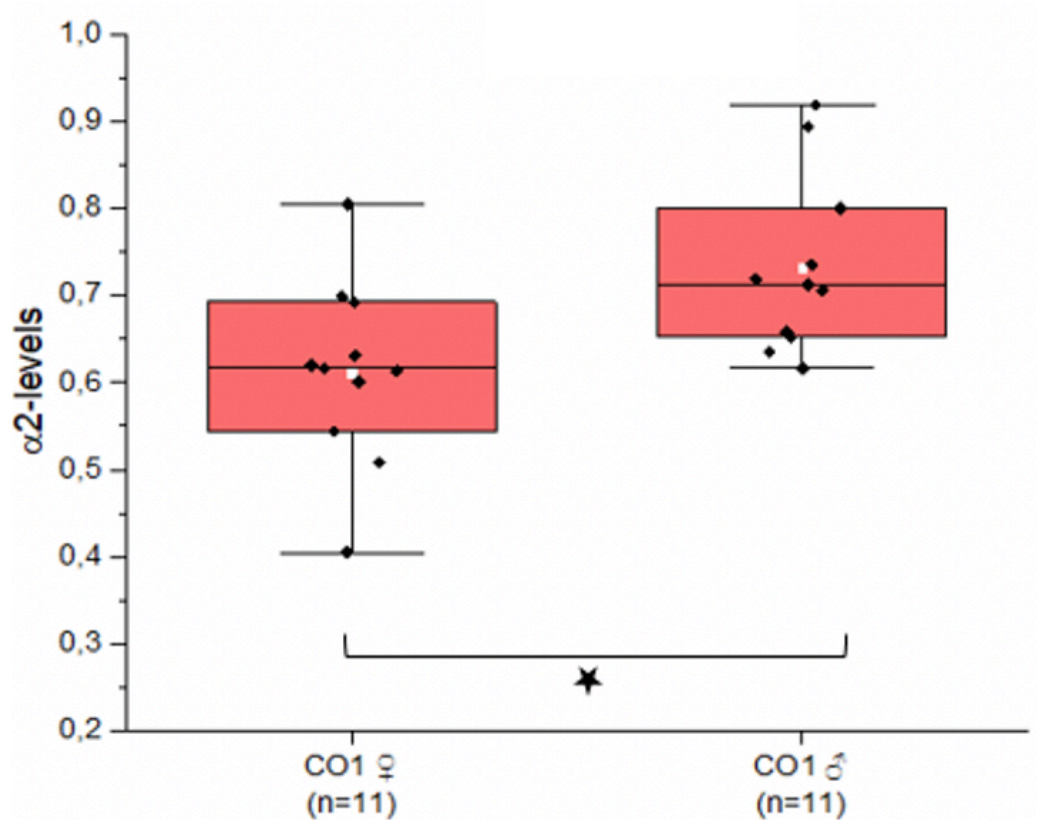

COI	α2 - Mean	SD	Transmission
**female (n=20)**	57.2	8.6	55%
**male (n=20)**	66.5	57.2	55%

Single Flies FG111
(α2, ß1) – *COI, Hind*III

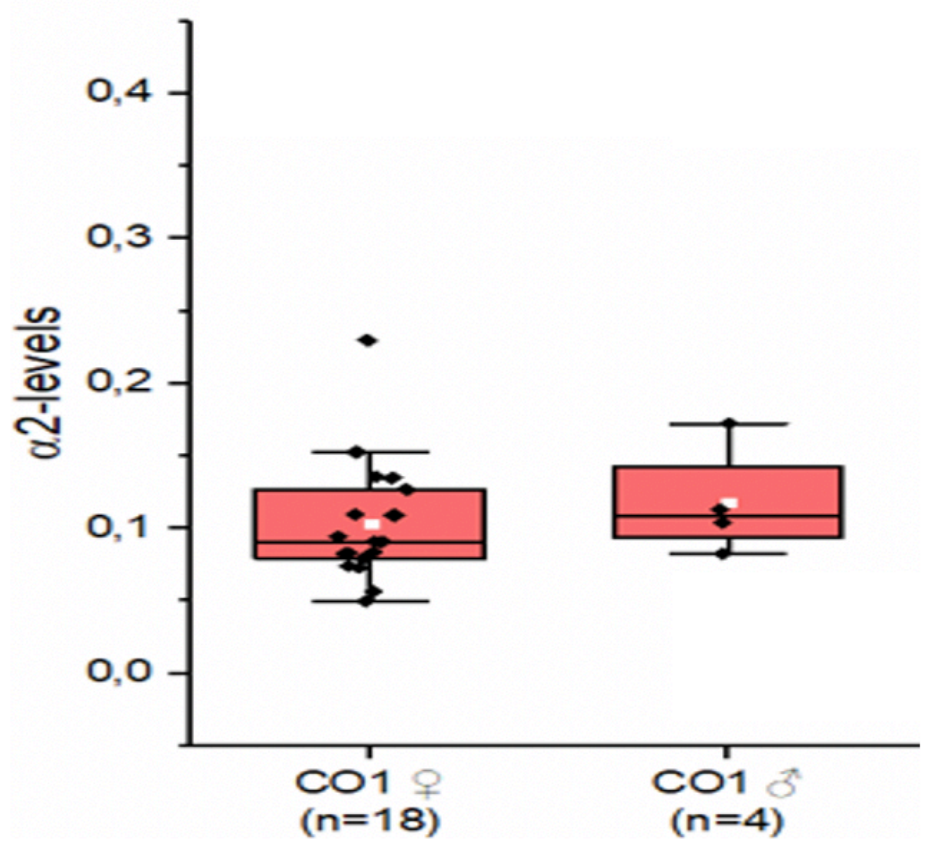

COI	α2 - Mean	SD	Transmission
**female (n=20)**	10.3	4.0	90%
**male (n=20)**	11.8	3.2	20%

Figure S1. Schematic illustration of Restriction Fragment Length Polymorphism (RFLP) for differentiating α from β mitotypes in *D. paulistorum*. (A) α-mitotype-specific RFLP-Southern blot. Whole fly DNA was fully digested with *Hin*dIII-enzyme, since only the α-mitotype displays the diagnostic SNP within the *CO1* restriction site (scissor), generating two 2.4 kb fragments, whereas the β-mitotype, lacking this restriction site at this position, thereby retains the 4.8 kb fragments. The internal *CO1*-probe of 788 bp cloned in a plasmid (green) covering the diagnostic SNP was used for their detection by Southern hybridization **(B)** Independent *CO1* Southern blot on *Hin*dIII digested DNA of C2 and A28 (each derived from a pool of 10 females) and 10 single fly O11 females probed with *CO1*. Equal DNA concentrations of app. 1µg was loaded per lane. Signal intensity differences between O11 samples represent quantitative differences in total DNA extracted from single flies. **(C)** Schematic

description of the α-diagnostic RFLP-PCR assay. Similar to RFLP-Southern blot, the same diagnostic *Hin*dIII SNP (scissor) was used. PCR from total DNA with the consensus primer 106 and 154 (arrows) in *CO1* give rise to a 788 bp fragment in both mitotypes (U). After digestion (D), the β-mitotype remains undigested due to the absence of the *Hin*dIII site (upper 788 bp band in D), but the α-mitotype is represented by two bands of 688 bp and 100 bp length. **(D)** Schematic description of the α1-diagnostic RFLP-PCR assay. Here, diagnostic *Eco*RI SNP (scissor) was used. PCR from total DNA with the consensus primer 106 and 154 (arrows) in *CO1* give rise to a 788 bp fragment in both mitotypes (U). After digestion (D), the β-mitotype remains undigested due to the absence of the *Eco*RI site (upper 788 bp band in D), but the α1-mitotype is represented by two bands of 455 bp and 333 bp length. (**E**) Representative genotyping of the Mega-NUMT positive lines shown in **Table 1** by two randomly picked females and males per line, *CO1* RFLP/*Hin*dIII*,* undigested (U) and digested (D). (**F**) Mega-NUMT genotyping via RFLP-PCR of four independent representative *D. paulistorum* isolines. For details see Material and Methods.

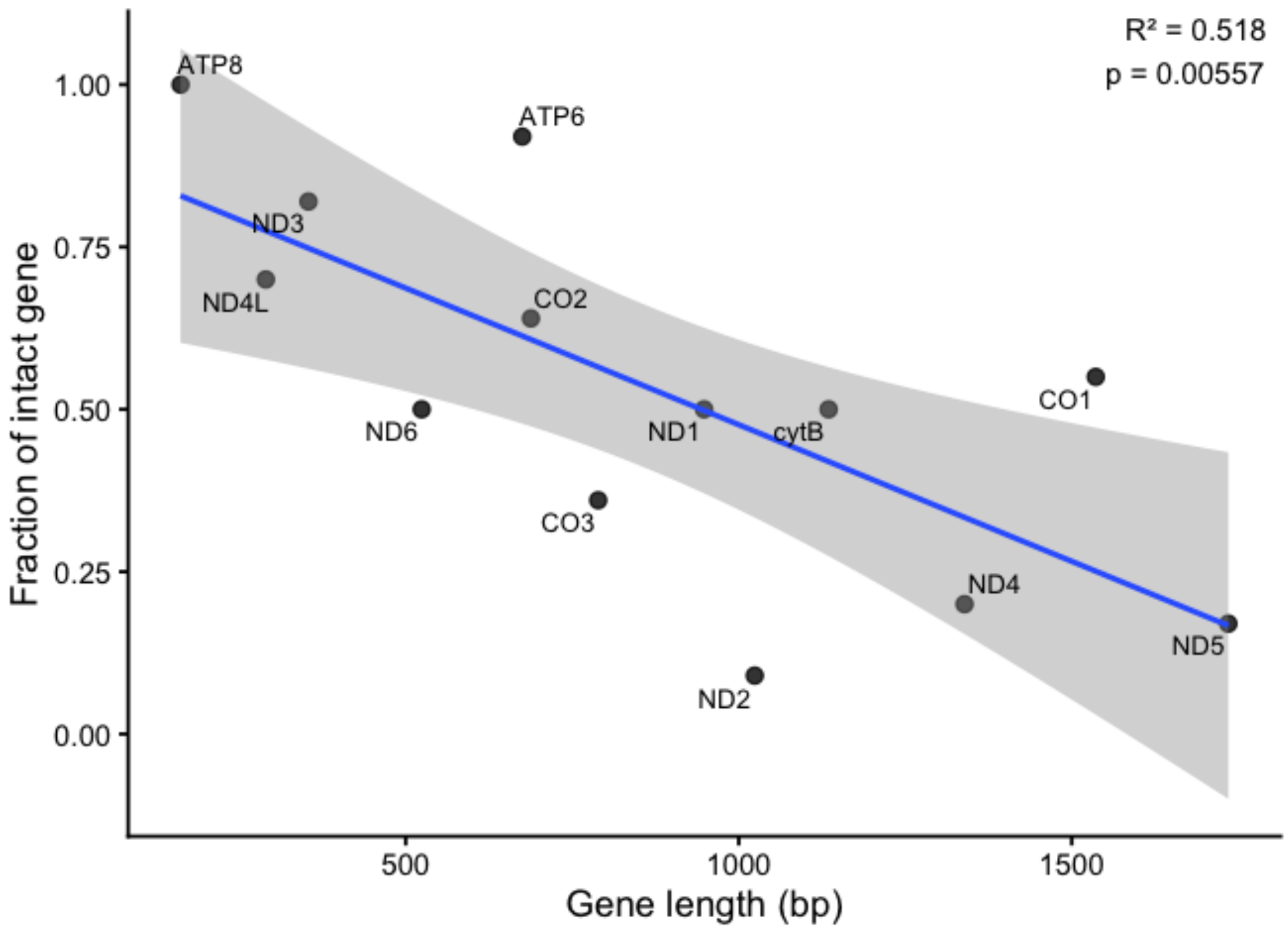

Figure S2. Relationship between gene length and fraction of intact gene across the 12 alpha-NUMT copies. Scatterplot showing gene length (bp) versus fraction of intact gene for individual genes. The fraction of intact genes was calculated as the number of copies with a pseudogenizing mutation divided by the number of copies present for each gene (see **Table S5**), and the gene length is the length of the intact gene. The solid line represents the linear regression fit, with the shaded area indicating the 95% confidence interval. R^2 and p-value are from a linear regression model testing the association between gene length and fraction intact.

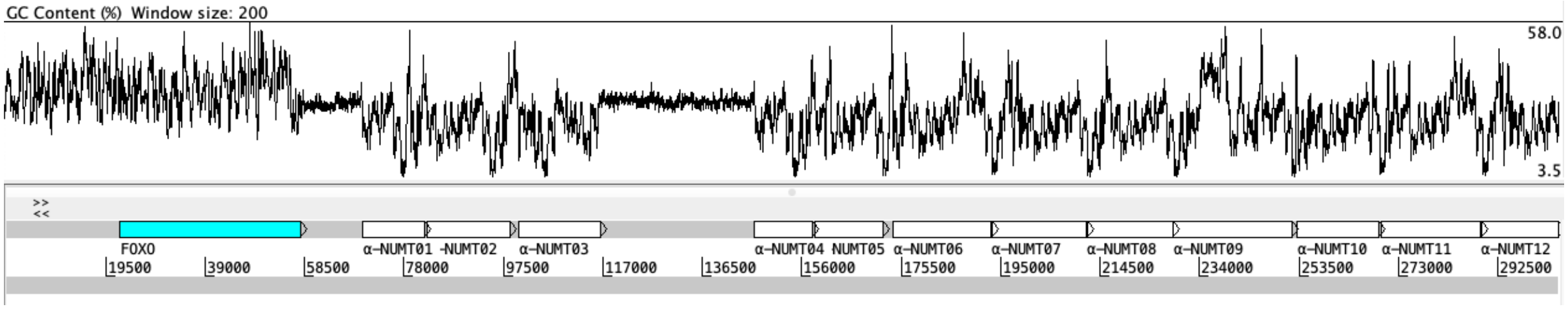

Figure S3. GC content (%) across the end region of contig000940. First base represents base 7.300.000 of the contig. Sliding window size for GC content values is 200. The nuclear gene *FOXO* is marked in blue. The α-NUMT copies are marked in white. GC values in the satellite sequences (between FOXO and α-NUMT01, and between α-NUMT03 and α-NUMT04) are low, with an average of 29.53% and 30.44% GC content, respectively, and very constant throughout the sequence. The average GC content throughout the nuclear genome assembly is 37.76 %, and the average GC content across the 12 α-NUMTs is 24.63%.

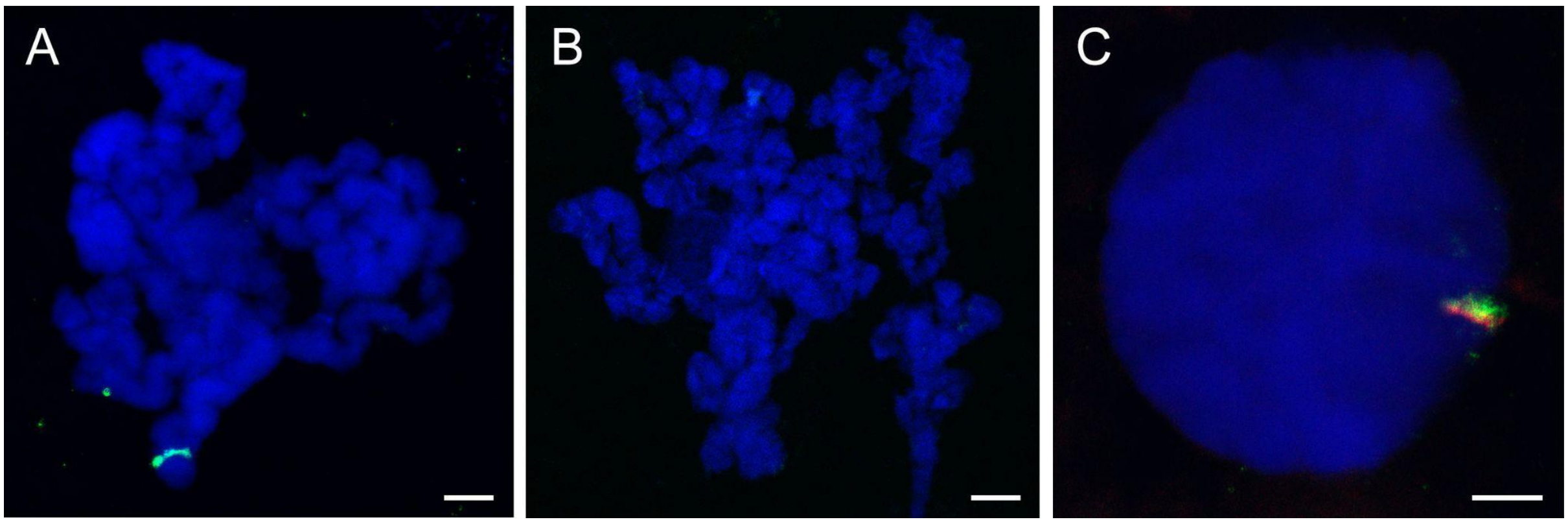

Figure S4. Localization of the Mega-NUMT in *D. paulistorum* O11 line chromosomes 3 using DNA FISH with mitochondria-specific probes. (A) Mega-NUMT band (green) on polytene chromosome 3 from larval salivary glands of the O11 line. Note the position of the band at the end of the chromosome arm. **(B)** The Mega-NUMT is absent in the FG295 line used as a control in the study. **(C)** Co-localization of the mega-NUMT (green) with the *FOXO* gene (red) in the salivary gland nucleus.

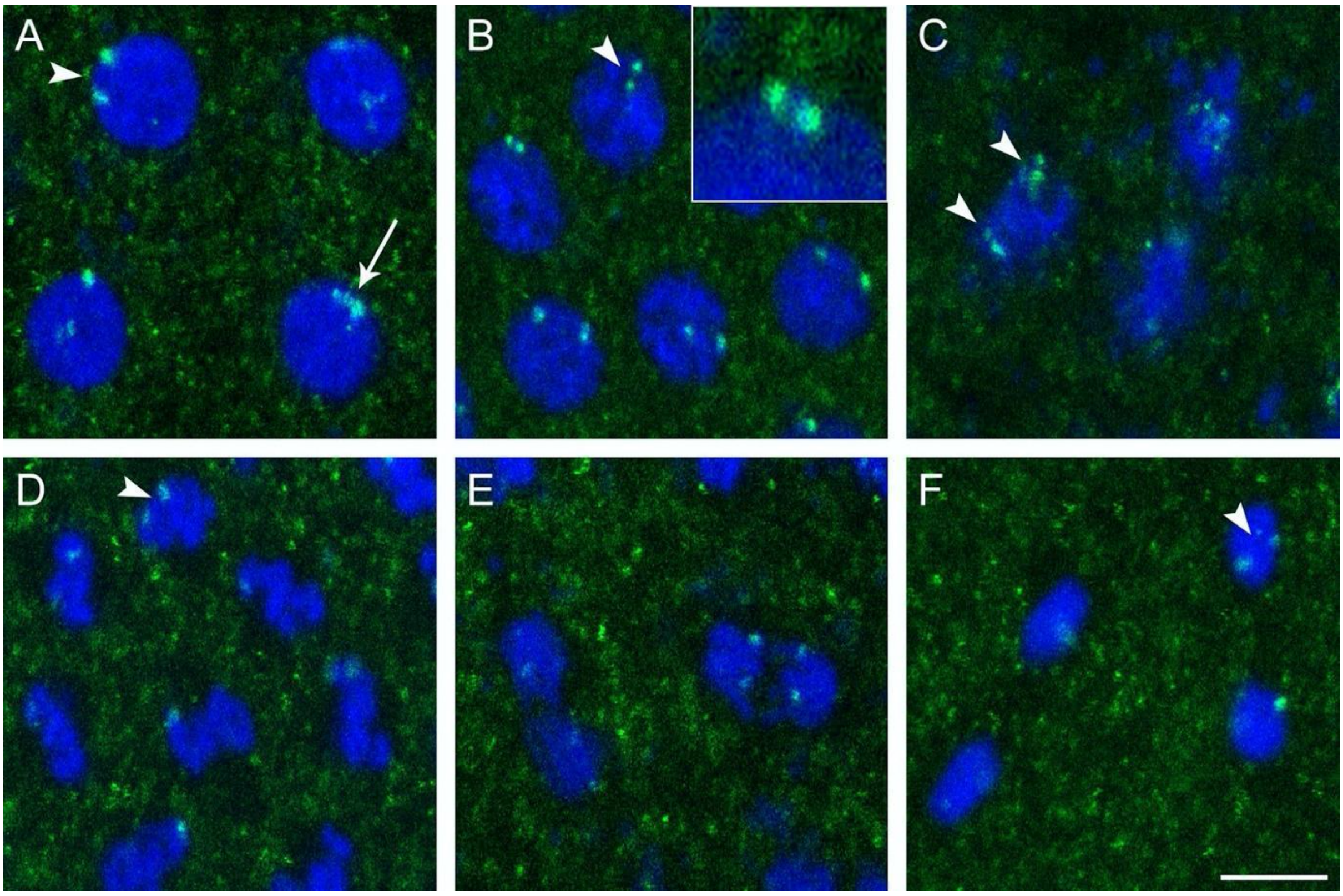

Figure S5. Mega-NUMT dynamics in blastodermal embryo mitosis. In mitotic nuclei at interphase **(A)**, prophase **(B)**, prometaphase **(C)**, metaphase **(D)** and telophase **(F),** the Mega-NUMT is observed mostly as a two-dot signal (green, arrowheads) with variegating distances between the dots. In some cases, the dots are fused into one big dot (green, arrow). In anaphase **(E)**, the Mega-NUMT signal is represented as 4 dots (2 dots per each segregating set of chromosomes). DNA is stained with DAPI (blue). Scale bar: 5 µm.

A

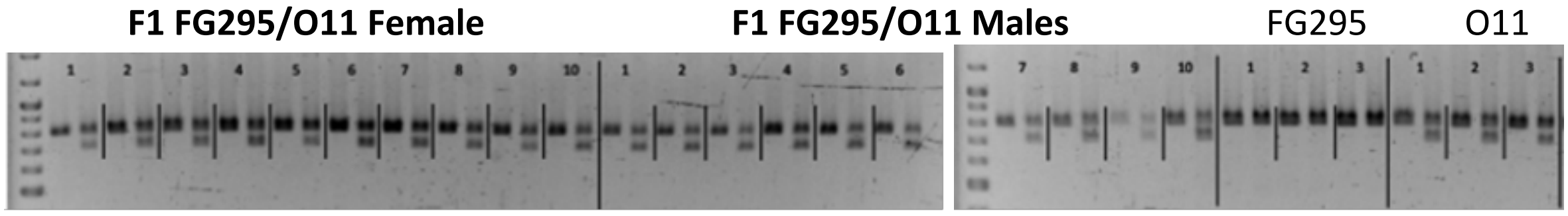

B

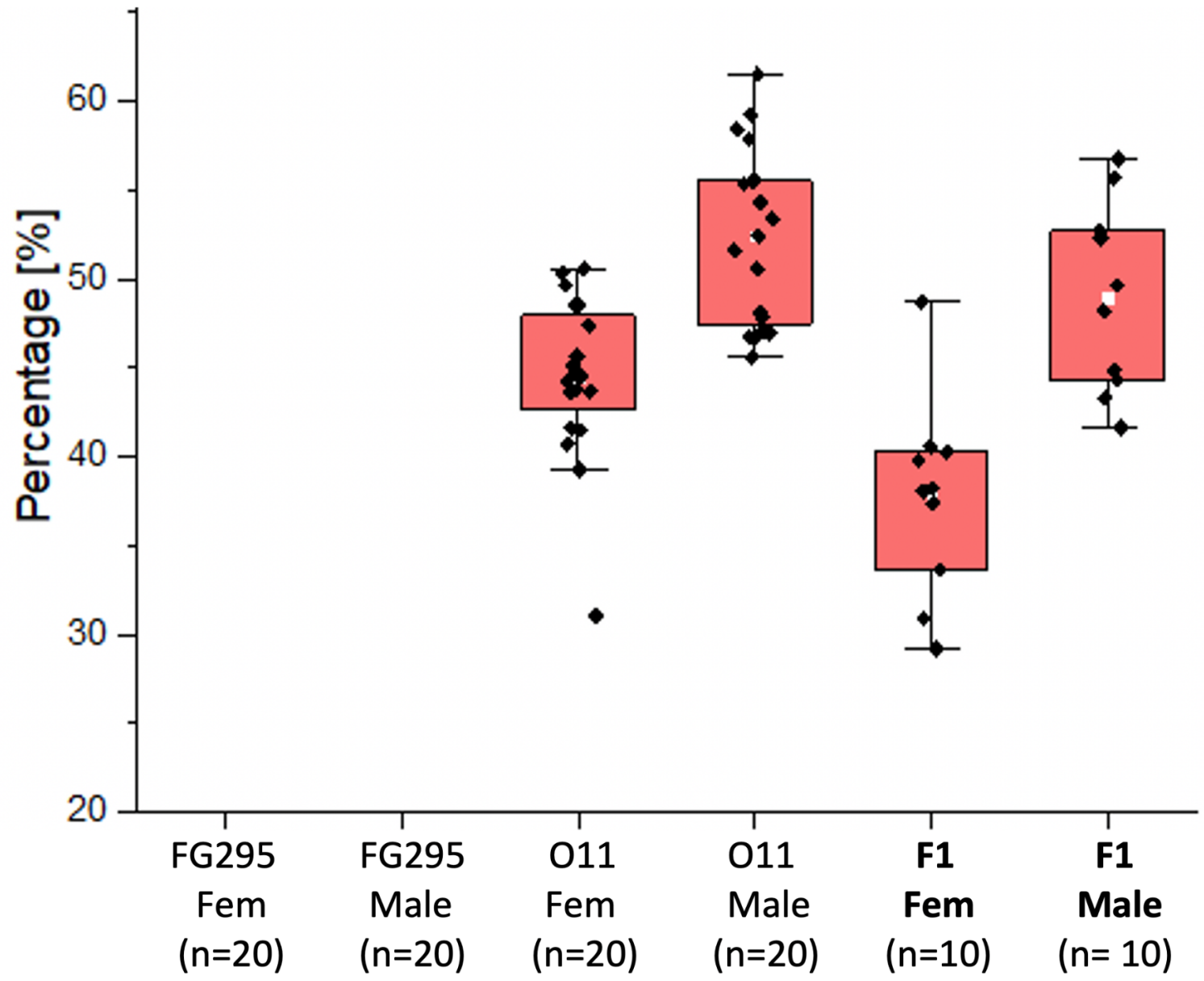

C

D. paulistorum line	Mean MN abundance	SD	Transmission rate
O11 females (n=20)	44.48	4.49	100%
O11 males (n=20)	52.47	4.84	100%
FG295 females (n=20)	0	0	0%
FG295 males (n=20)	0	0	0%
**F1 females (n=10)**	**37.67**	**5.54**	**100%**
**F1 males (n=10)**	**48.94**	**5.35**	**100%**

Figure S6. Perfect transmission of the *Dpau* Mega-NUMT (MN) in F1 single fly hybrids (bold) of FG295 (MN-negative) and O11 (MN-positive) parents. (**A**) *CO1* RFLP/*Hin*dIII-PCR of female and male F1 hybrids (bold) and their parental lines (for technical details see **Fig. S1**). (**B**) MN signal quantification of the *Hin*dIII sensitive band in individual flies. (**C**) Perfect transmission rate in F1s (n=20) confirming Mega-NUMT homozygosity in the O11 line of *D. paulistorum*.

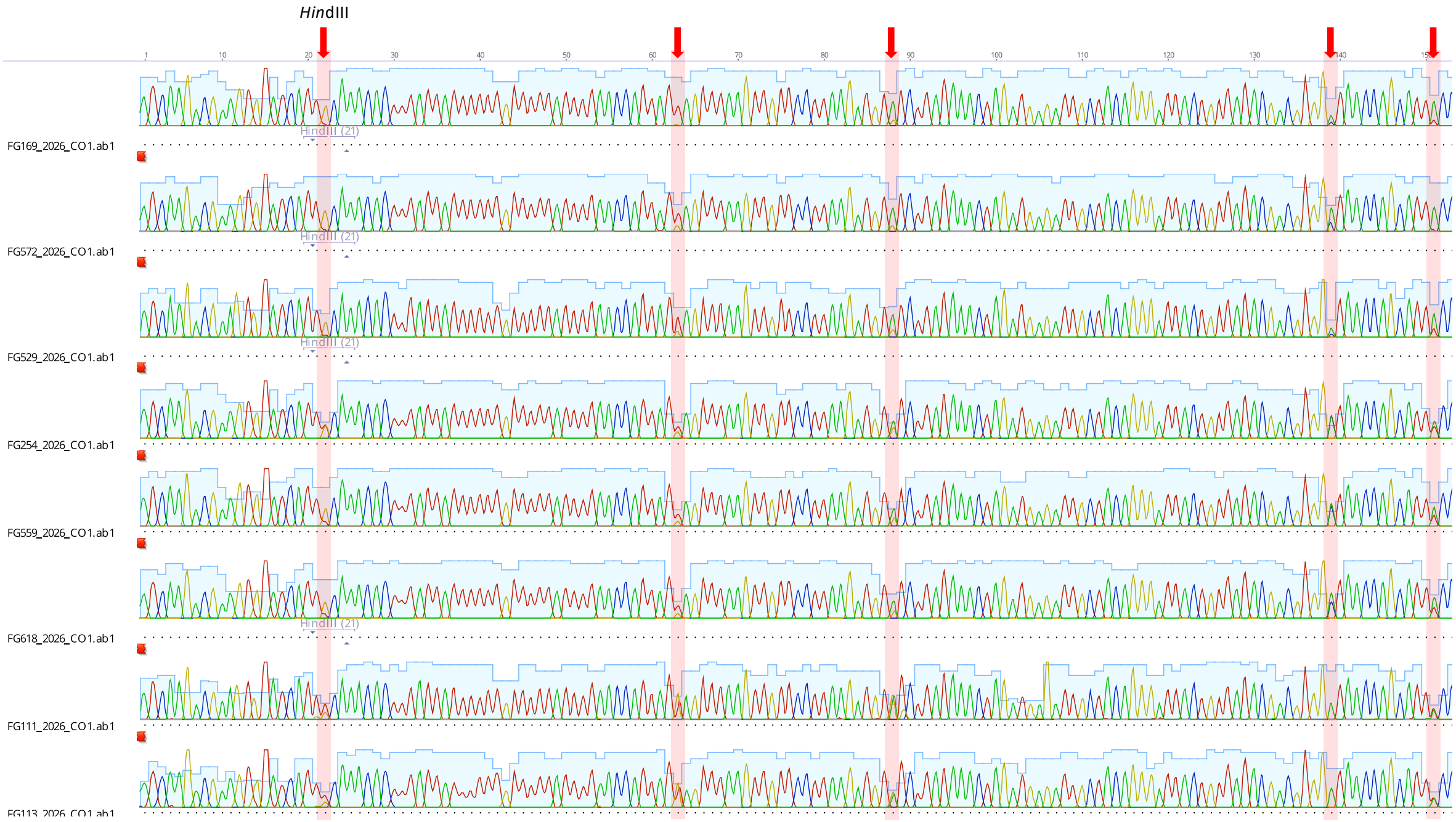

Figure S7. Example of aligned *CO1* chromatograms of Mega-NUMT carrying candidate isolines from French Guiana by direct Sanger re-sequencing in 2026. Polymorphic sites in *CO1* indicating the presence of the Mega-NUMT of *D. paulistorum spp.* by double-peeks (red arrow and highlighted in pink) and their diagnostic *Hin*dIII restriction site of isolines that have persisted under lab conditions from 2014 to 2026.
